# Every 1,000 steps matter: incremental reductions in metabolic syndrome risk in Japanese office workers

**DOI:** 10.1186/s13098-025-01816-3

**Published:** 2025-07-18

**Authors:** Yukako Yamaga, Thomas Svensson, Ung-il Chung, Akiko Kishi Svensson

**Affiliations:** 1 Precision Health, Department of Bioengineering, Graduate School of Engineering, The University of Tokyo, Tokyo, 113-8656 Japan; 2 Graduate School of Health Innovation, Kanagawa University of Human Services, Kawasaki, 210-0821 Japan; 3 Department of Clinical Sciences Malmö, Lund University, Skåne University Hospital, CRC, Jan Waldenströms Gata 35, Malmö, 205 02 Sweden; 4 Clinical Biotechnology, Center for Disease Biology and Integrative Medicine, Graduate School of Medicine, The University of Tokyo, Tokyo, 113-8656 Japan; 5 Department of Diabetes and Metabolic Diseases, The University of Tokyo, Tokyo, 113-0033 Japan

**Keywords:** Incidence, Metabolic syndrome, Physical activity, Prospective, Step count, Wearable device

## Abstract

**Background:**

Several studies have investigated the association between metabolic syndrome (MetS) and physical activity (PA). However, further research is needed using objective measures of PA in free-living conditions, while also accounting for the time-variant nature of MetS. This study aimed to: (1) investigate the association between wearable device-measured step count and 5-year MetS incidence in generally healthy Japanese participants, using annual health check-up (AHC) data and interval-censored survival analysis; and (2) assess the current, and recently revised, reference value (8,000 steps/day) of Japan’s Ministry of Health, Labour and Welfare (MHLW), and (3) investigate the possibility of non-linear associations between daily step count and MetS.

**Methods:**

This longitudinal prospective observation study identified average daily step count per year/person as the main exposure, and MetS incidence, defined according to Japanese guidelines, as the main outcome. The main analysis included 730 participants without MetS or pre-MetS at baseline. An interval-censored Cox model was applied to assess MetS incidence using time-to-event data.

**Results:**

Every 1,000 steps added to the average step count was significantly and inversely associated with incident MetS in adjusted models [Model 1: HR = 0.92; 95% CI: 0.85, 1.00; Model 2: HR = 0.91; 95% CI: 0.83, 0.99] (*p* < 0.05). The current reference value (8,000 steps/day) also indicated a significant inverse association [Model 1: HR = 0.32; 95% CI: 0.14, 0.71; Model 2: HR = 0.26; 95% CI: 0.11, 0.61] (*p* < 0.01). Higher daily step counts lowered the risk of MetS incidence according to the amount of steps up to a step count of 12,000, however, the dose-response effect was attenuated beyond 12,000 steps/day.

**Conclusions:**

Each additional 1,000 daily steps was associated with a 9% reduction in the risk of developing MetS among healthy participants. The reference value (8,000 steps/day) was associated with a 74% reduction in MetS risk.

## Introduction

Metabolic syndrome (MetS) is known as a cluster of health conditions associated with hyperglycemia, insulin resistance, dyslipidemia, and hypertension which may lead to future diseases such as cardiovascular disease (CVD) and type 2 diabetes mellitus [1, 2]. Although the pathogenesis of MetS has not been fully elucidated, it is believed to involve a combination of biological factors, and individual lifestyle factors such as dietary habits and physical activity (PA). In recent years, MetS has garnered increasing public health attention due to its rising global prevalence, as recognized by various health organizations despite differing diagnostic criteria [3, 4], and its potential impact on future medical expenditures. In response, health organizations have promoted awareness and implemented policies aimed at reducing the incidence of lifestyle-related non-communicable diseases (NCDs). In Japan, the Japanese National Health Screening and Intervention Program, implemented in April 2008, mandates that health insurers provide specific health programs that aim for the early detection of MetS among insured individuals aged 40–74 years [5]. Based on their individual risk profiles, participants receive either motivational or intensive health guidance from health professionals with follow-up consultations spanning 3 to 6 months.

Previous studies, including our own work, have shown that PA is inversely associated with MetS [6–8]; and further that adequate PA leads to a reduction in risk of all-cause mortality [9, 10]. However, the extent to which PA contributes to a reduction in MetS incidence, or how time-dependent changes influence MetS development, remains unclear. Many previous studies have relied on self-reported questionnaires to assess daily PA, which introduces the potential for recall bias [11]. Furthermore, some longitudinal studies that objectively measured PA using pedometers or accelerometers did so only over brief periods at the beginning of the study [7, 9, 12], limiting their ability to assess long-term patterns in daily PA, particularly given that health behaviors may fluctuate over time. Another challenge lies in establishing a persuasive study period and appropriate research design [8]. Lifestyle interventions requiring long-term follow-up of disease incidence are costly, and because MetS develops gradually, it is difficult to determine the precise time of onset. Furthermore, step-based PA has recently been proposed by health organizations as health target for the general population, owing to its practicality and clear measurability. In Japan, the recommended PA threshold has been updated [13]; however, the recommended number of daily steps varies across health organizations [10]. Some studies report that the dose-response benefit may diminish beyond a certain step count, while others suggest that higher daily step counts are linearly and inversely associated with health risks [7, 10, 12].

Considering these factors, gaining greater insight into PA under free-living conditions may enhance current public health strategies. Accordingly, this study aimed to: (1) investigate the association between wearable-measured daily step count under free-living conditions and 5-year incidence of MetS in healthy participants using annual health check-up data; (2) evaluate the effectiveness of the current PA guideline of 8,000 steps/day, as recommended by the Ministry of Health, Labour and Welfare (MHLW) in Japan [13]; and (3) explore potential non-linear associations between daily step count and MetS [7, 12].

## Methods

### Study design and study population

This study was conducted as a prospective observational study. Data from annual health check-ups and wearable devices were provided by a health insurance company in Tokyo, Japan, which had implemented a behavioral change program for its full-time employees. The data collection period spanned from April 1, 2016, to December 31, 2020. Informed consent was obtained from all participants prior to the study, following a detailed explanation of its purpose. Participation eligibility was not dependent on enrollment in the behavioral change program. The study was approved by the Ethics Committee of the School of Engineering, the University of Tokyo (Approval No.: KE19-14).

**Fig. 1:**
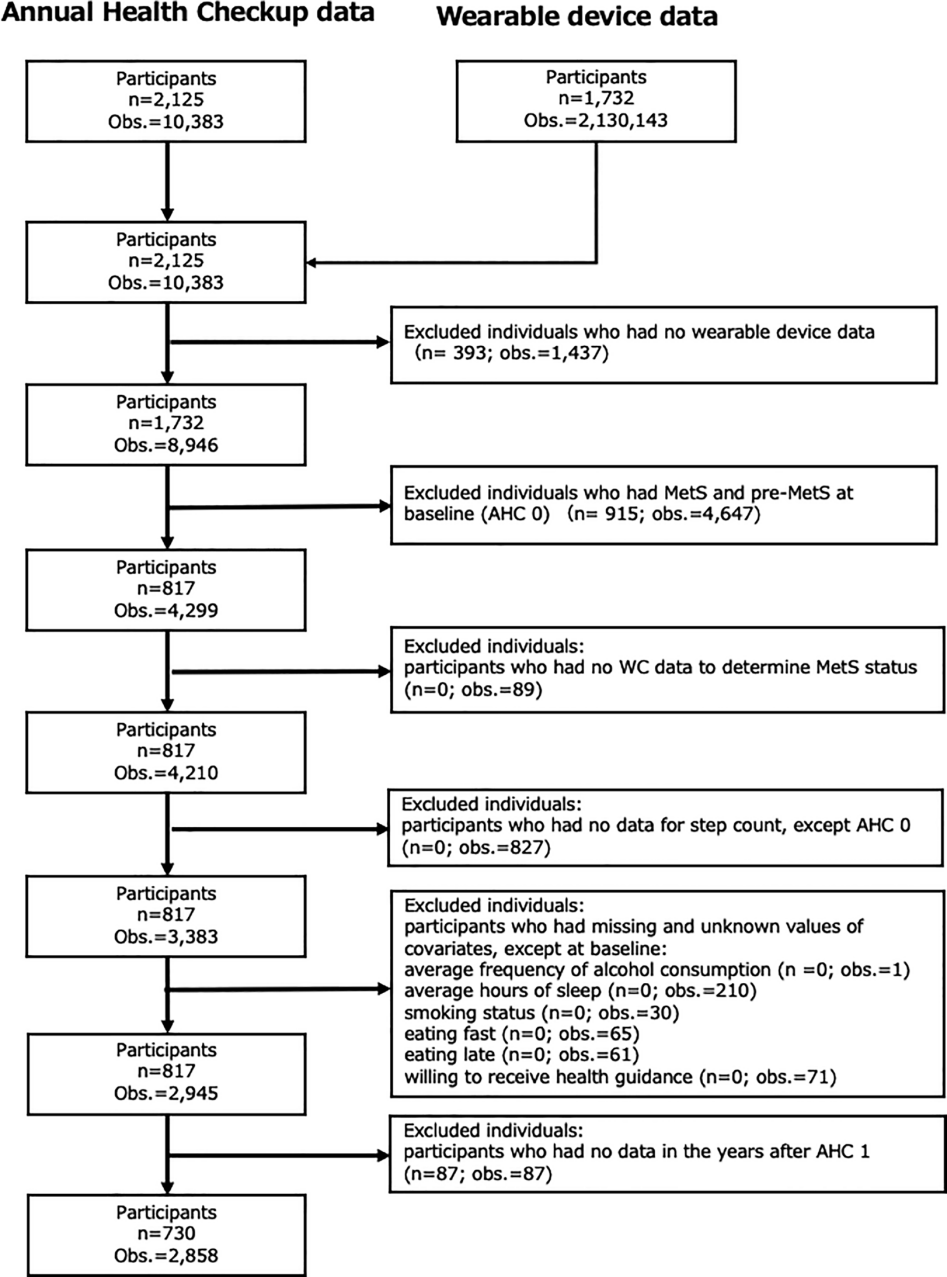
Flowchart for the main analysis detailing the inclusion and exclusion of participants and observations. Abbreviations: obs: observations. MetS: metabolic syndrome, pre-MetS: preliminary status of MetS, AHC: Annual Health Check-up, WC: waist circumference

The wearable device dataset included 1,732 participants with a total of 2,130,143 observations. Previous studies suggests that even sedentary individuals typically accumulate approximately 5,000 steps/day [10], and values below 1,000 steps/day are commonly interpreted as indicative of non-wear days or minimal device use; accordingly, such values have been used as exclusion criteria in wearable-based research [14]. For total sleep time, values below 120 min per 24-hour period were treated as missing values. While the manufacturer’s algorithm for sleep detection is not publicly disclosed, prior validation studies have reported individual-level discrepancies of approximately 2 hours between wearable devices and gold-standard polysomnography [15]. Additionally, daytime naps lasting 1–3 hours may be distinguished from nighttime sleep [16]. Based on these characteristics of real-world wearable data, PA values ≥ 1,000 steps/day and total sleep time ≥ 120 min/24-hour period were treated as valid and biologically plausible. Valid daily records were used to calculate intra-individual annual averages for step count and total sleep time, which were then merged with annual health check-up (AHC) data for subsequent analysis. Using pseudonymised participant IDs, individuals present in both the wearable device dataset and AHC dataset were included, while those without wearable device data were excluded (*n* = 393; 1,437 observations) (Fig. 1). To meet the primary objective, participants with prevalent MetS and pre-MetS at the first annual health check-up (AHC 0), were removed from the dataset (*n* = 915; 4,647 observations). Participants with insufficient information on waist circumference (WC) during the study period were excluded, as this prevented classification of the main outcome (*n* = 0; 89 observations). Observations with missing values for the main exposure during the follow-up period, were excluded (*n* = 0; 827 observations). Observations were excluded for participants with missing values on the average frequency of alcohol consumption (*n* = 0; 1 observation), average hours of sleep, including unreasonable values (*n* = 0; 210 observations), smoking status (*n* = 0; 30 observations), dietary behaviors such as “eating faster than others” (*n* = 0; 65 observations) and “having an evening meal within 2 hours before bedtime” (*n* = 0; 61 observations), and health motivation (*n* = 0; 71 observations). Of the 817 participants remaining, those who provided AHC information only at the baseline examination (AHC 0) but had no follow-up data in subsequent years were excluded (*n* = 87; 87 observations). Finally, 730 participants (435 men and 295 women) with 2,858 observations were included in the main analysis, each contributing between one and five annual observations over the 5-year study period, excluding AHC 0.

### Data collection

#### Wearable device for physical activity data

PA data were obtained using a consumer wearable device widely distributed in Japan: the Fitbit (Fitbit Inc., San Francisco, CA, USA, https://www.fitbit.com). The device has been used in several studies to measure sleep time and PA level as part of real-world data collection [17, 18]. Data from the Fitbit are synchronized with a smartphone via Bluetooth when an internet connection is available, allowing daily measurements of each user’s step count. The company provided the Fitbit devices at its own expense as part of a health management campaign and instructed employees to wear them around the clock, except during bathing or battery charging.

#### Health data from annual health check-up data

Study participants included in the analysis had records from their first annual health check-up (AHC 0) and at least one follow-up AHC record, allowing observation of changes in MetS status over time. AHC data included two types of individual-level information: (1) anthropometric and biochemical measurements, and (2) responses to general lifestyle questionnaires. Biochemical examinations included the required items for MetS classification, such as triglycerides (TG), high-density and low-density lipoproteins (HDL, LDL), blood pressure, fasting blood glucose (FBG) and hemoglobin A1c (HbA1c), while anthropometric measurement of WC was performed by healthcare professionals. The questionnaires assessed general lifestyle behaviors, including sleep, smoking, alcohol consumption, dietary habits, and exercise, with participants instructed to respond based on their status during that year.

#### Metabolic syndrome status (main outcome)

The main outcome of this study was the incidence of MetS among participants who were classified as having no MetS or pre-MetS at baseline (AHC 0). MetS status was categorized into 3 groups (no MetS, pre-MetS, and MetS) based on AHC data, in accordance with the 2005 criteria of the Japanese Society of Internal Medicine [19]. MetS was defined by the presence of one obligatory criterion - WC ≥ 85 cm in men and ≥ 90 cm in women – combined with at least two of the following non-obligatory criteria: (1) systolic blood pressure (SBP) ≥ 130 mmHg and/or diastolic blood pressure (DBP) ≥ 85 mmHg; (2) TG ≥ 150 mg/dL and/or high-density lipoprotein cholesterol (HDL-C) < 40 mg/dL; and (3) FBG ≥ 110 mg/dL. Participants who met the obligatory criterion and only one of the non-obligatory criteria were classified as having pre-MetS, while those who did not meet the obligatory criterion for WC were classified as having no MetS. A binary outcome variable was created to indicate MetS incidence (0 = did not develop MetS, 1 = developed MetS).

#### Physical activity (main exposure)

The main exposure was daily step count, as measured by the wearable device. Intra-individual annual averages for daily step count and total sleep time were calculated using the original panel dataset, which included multiple daily records per participant. The average number of valid days per participant per year was 162 for step count and 103 for total sleep time. Previous studies have reported that each additional 1,000 steps per day is associated with a reduction in all-cause mortality [9, 20]. Accordingly, average daily step count was standardized in 1,000-step units to represent incremental increases. In line with current national guidelines in Japan, MHLW issued the updated Exercise and Physical Activity Guide for Health Promotion in 2023, revising the earlier Exercise and Physical Activity Reference for Health Promotion (EPAR) published in 2006 and 2013 [21]. The updated guide recommends 8,000 steps per day or 60 min per day of moderate to vigorous physical activity (MVPA) for adults, which is equivalent to 23 metabolic equivalents (METs) h/week [13]. To assess adherence to the recommended step count, the proportion of valid days per year on which each participant achieved ≥ 8,000 steps was calculated. These proportions were averaged across available years to represent each participant’s annual achievement rate. To explore the possibility of a non-linear association between daily step count and MetS, annual average daily step count was categorized into five groups: <5,000; ≥5,000 to < 8,000; ≥8,000 to < 10,000; ≥10,000 to < 12,000; and ≥ 12,000 steps per day.

### Covariates

Covariates associated with the main outcome and exposure were obtained from AHC records and wearable device data and included: (1) individual characteristics: sex and age; (2) lifestyle factors: average frequency of alcohol consumption (never or rare/sometimes/everyday) [22]; average hours of sleep from the wearable device [23]; current smoking status (non-smoker/smoker) [24]; and dietary behaviors [25], including “eating faster than others” (slower/general/faster) [26] and “having an evening meal within two hours before bedtime more than three times per week” (yes/no) [27]; and (3) motivation to receive health guidance from healthcare professionals, if necessary (yes/no). In the original dataset, 57% of alcohol consumption frequency observations were missing; to address this, a mean value was calculated for each participant across the three response categories (never or rare/sometimes/everyday) over the study period. This average was then used to construct a variable representing each individual’s propensity for alcohol consumption during the study period.

### Statistical analysis

The dataset was structured with multiple records per participant, incorporating time-varying covariates and follow-up intervals calculated in days from baseline. Interval-censored Cox proportional hazards regression models were used to estimate hazard ratios (HR) and 95% confidence intervals (CI) for the association between daily step count and incident MetS, defined as the onset of MetS or pre-MetS. Because the exact timing of the event was unknown, the AHC at which a participant’s MetS status changed was used to define the event interval. Observations were treated as right-censored if MetS did not develop during the study period, and as interval-censored if MetS developed between consecutive AHCs. In addition to the unadjusted model, Model 1 included age and sex, while Model 2 further adjusted for lifestyle factors (average frequency of alcohol consumption, average hours of sleep, smoking status, and dietary behaviors), and health motivation (willingness to receive health guidance if necessary). To assess model fit and the possibility of a non-linear association between daily step count and MetS incidence, restricted cubic spline models with three knots at 10th, 50th, and 90th percentiles, and were fitted using interval censored Cox regression. Step count was centered around the sample mean (approximately 9,500 steps/day) prior to spline transformation to facilitate interpretation. The non-linear (spline-based model) was compared to a linear model using Akaike’s Information Criterion (AIC), Bayesian Information Criterion (BIC), and a likelihood ratio test to determine whether the more complex model provided improved explanatory power. The linear model demonstrated a better fit and was therefore retained for the main analyses. Additionally, the cumulative probability of developing MetS was visualized using Kaplan-Meier curves based on Model 2. All analyses were conducted using Stata/MP version 18.5 (StataCorp LLC, College Station, TX, USA; https://www.stata.com/company/).

### Sensitivity analysis with different MetS classification

In addition to the main analysis, which assessed incident MetS defined as the onset of MetS or pre-MetS, an alternative classification was tested to enable comparison with the results of the main analysis. In this sensitivity analysis, pre-MetS at baseline, along with no MetS, was classified as a healthy status. Participants with MetS at baseline were excluded from the initial dataset (*n* = 809; 4,112 observations). Observations with unknown values for the main outcome (*n* = 0; 96 observations) and the main exposure (*n* = 0; 939 observations) were excluded. Missing values for covariates were also excluded, including: average frequency of alcohol consumption (*n* = 0; 1 observation); average hours of sleep (*n* = 0; 227 observations), smoking status (*n* = 0; 31 observations); dietary behavior of “eating faster than others” (*n* = 0; 81 observations) and “having an evening meal within two hours before bedtime” (*n* = 0; 69 observations); and health motivation (*n* = 0; 78 observations). Participants with no follow-up data after AHC 1 were excluded (*n* = 96; 96 observations). As a results, 827 participants (528 men and 299 women) with 3,216 observations were included in the sensitivity analysis. The main outcome was treated as a binary variable (0 = did not develop MetS; 1 = developed MetS).

## Results

### Main analysis

**Fig. 2:**
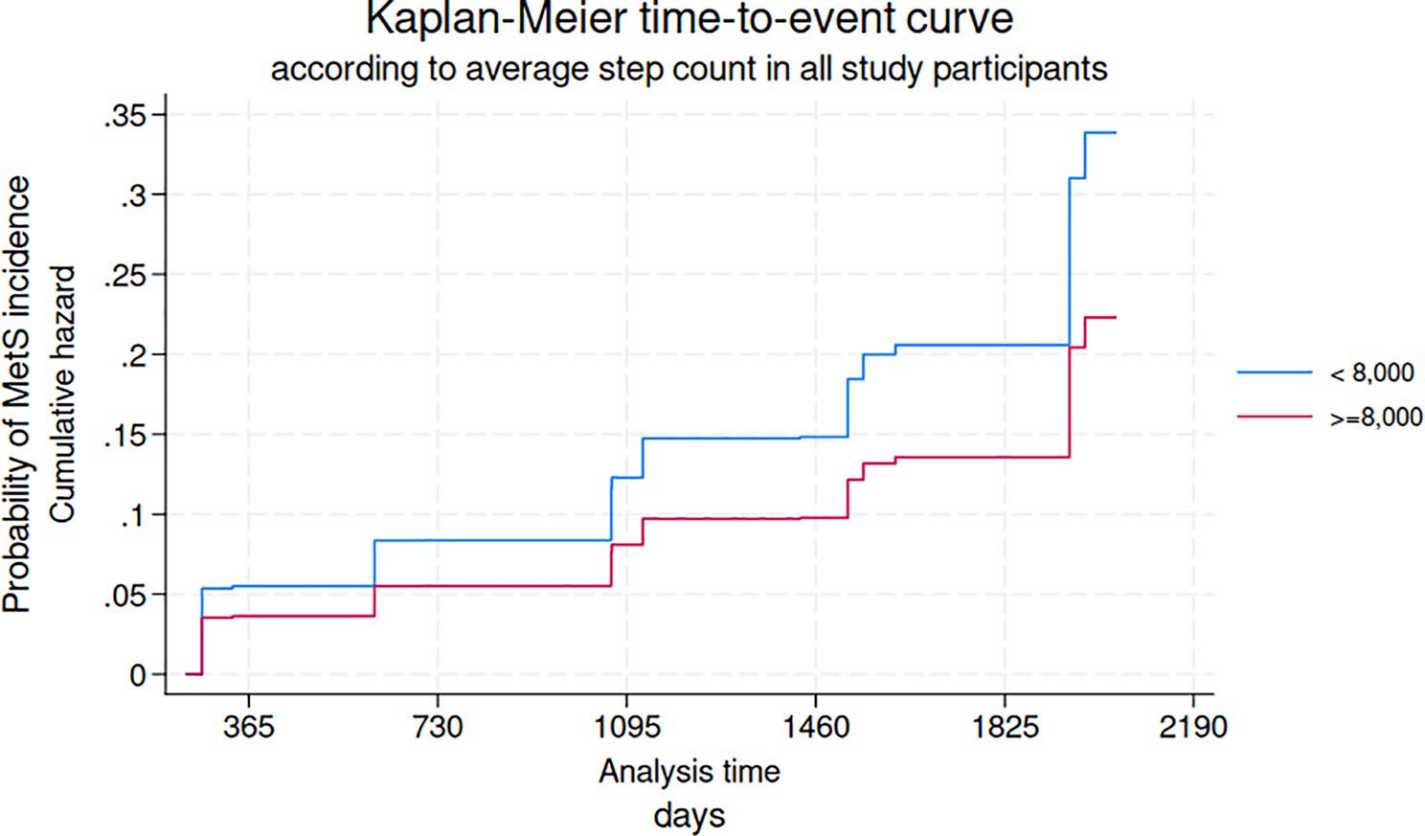
Kaplan-Meier time-to-event curve according to step count level in all study participants (*n* = 730). Kaplan-Meier time-to-event curve estimating the cumulative probability of incident metabolic syndrome (MetS & pre-MetS) according to the step count category (< 8,000 vs. ≥8,000 steps/day) among healthy participants, based on the fully adjusted model (Model 2) (*n* = 730). Abbreviations: MetS: metabolic syndrome

**Fig. 3:**
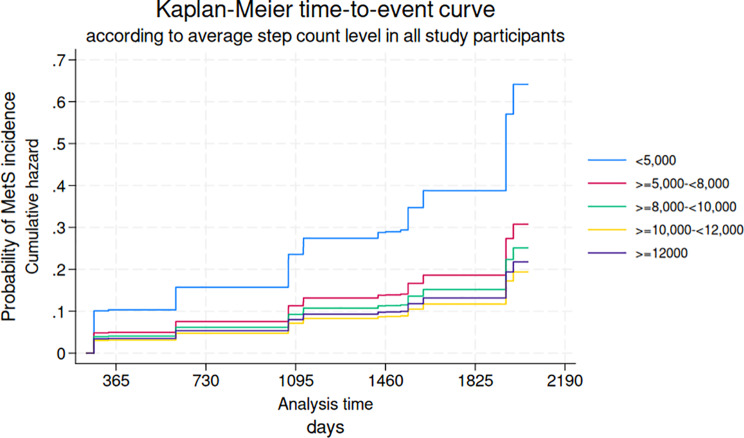
Kaplan-Meier time-to-event curve according to step count level in all study participants (*n* = 730). Kaplan-Meier time-to-event curve estimating the cumulative probability of incident metabolic syndrome (MetS & pre-MetS) by step count category among healthy participants, based on the fully adjusted model (Model 2) (*n* = 730). Abbreviations: MetS: metabolic syndrome

During the study period from AHC 0 to the final AHC for each participant (2015–2020), the mean follow-up time was 4.6 (± 1.2) years, totaling 2,624 person-years. Table 1 presents the baseline characteristics of the study population stratified by step count level at AHC 1. Table 2a shows that each 1,000-step increment was significantly and inversely associated with MetS incidence in all models except the unadjusted model [Unadjusted: HR = 0.97; 95% CI: 0.90, 1.06; Model 1: HR = 0.92; 95% CI: 0.85, 1.00; Model 2: HR = 0.91; 95% CI: 0.83, 0.99]. Table 2b presents the results for the current reference value (8,000 steps/day), showing a significant inverse association between daily step count and MetS incidence in all except unadjusted model [Unadjusted: HR = 0.64; 95% CI: 0.29, 1.40; Model 1: HR = 0.32; 95% CI: 0.14, 0.71; Model 2: HR = 0.26; 95% CI: 0.11, 0.61]. Table 2c reports HRs for average daily step count categories, showing significant inverse associations for all groups except the 5,000 to 8,000 steps/day group, compared with the referent < 5,000 steps/day [Model 2: ≥5,000 to 8,000: HR = 0.48; 95% CI: 0.18, 1.24; ≥8,000 to 10,000: HR = 0.39; 95% CI: 0.16, 0.98; ≥10,000 to 12,000: HR = 0.30; 95% CI: 0.12, 0.78; ≥12,000: HR = 0.34; 95% CI: 0.13, 0.89, respectively]. Figure 2 illustrates the cumulative probability of MetS incidence by step count category (< 8,000 vs. ≥ 8,000 steps/day). Risk was consistently higher among participants with < 8,000 steps/day, with the difference between groups increasing over time to approximately 1.5-fold by the fifth year. Figure 3 shows the cumulative probability of MetS incidence across step count categories among healthy participants, indicating that the group with the lowest daily step count had the highest risk.

**Table 1:** Characteristics of participants by average step count per year at AHC 1 (*n* = 730)

	Average step count per year	
	< 8,000 (*n* = 195)	≥ 8,000 (*n* = 535)
Men, n (%)	87 (44.6)	348 (65.1)
Age, median (iqr)	44 (40–49)	45 (41–51)
**Lifestyle factors**		
Alcohol frequency, n. (%)		
Rarely/Never	68 (34.9)	113 (21.1)
Sometimes	79 (40.5)	229 (42.8)
Every day	48 (24.6)	193 (36.1)
Average hours of sleep, median (iqr)	6.3 (5.3–7.3)	5.97 (5.4–6.5)
Smoking status, n (%)		
Non-smoker	152 (78.0)	423 (79.1)
Current smoker	43 (22.0)	112 (20.9)
Diet (eating faster than others), n (%)		
Slower	26 (13.3)	56 (10.5)
General	108 (55.4)	296 (55.3)
Faster	61 (31.3)	183 (34.2)
Diet (having evening meal within 2 h before bed), n (%)		
No	132 (67.7)	299 (55.9)
Yes	63 (32.3)	236 (44.1)
**Health motivation**		
Intention to receive health guidance if necessary, n (%)		
No	114 (58.5)	301 (56.3)
Yes	81 (41.5)	234 (43.7)
**Step count**		
Number of steps/day per year, median (iqr)	7,030 (6,079 − 7,533)	10,216 (9,046 − 11,693)
Average percentage achieving the MHLW reference value, median (iqr)^a^	11% (4–21%)	44% (23–60%)

Abbreviations: AHC: Annual Health Check-up, iqr: interquartile range, MHLW: Ministry of Health, Labour and Welfare

^a^ Proportion of valid days per year with ≥ 8,000 steps, averaged per participant across study years

**Table 2a:** Hazard ratios for incidence of metabolic syndrome risk in Japanese office workers (*n* = 730)

Model		HR (95% CI)	*P* value
Unadjusted	Average daily Step Count (per 1,000)	0.97 (0.90, 1.06)	0.54
Model 1^a^	Average daily Step Count (per 1,000)	0.92 (0.85, 1.00)	0.04
Model 2^b^	Average daily Step Count (per 1,000)	0.91 (0.83, 0.99)	0.02

**Table 2b:** Hazard ratios by achievement rate of the MHLW reference value in Japanese office workers (*n* = 730)

Model		HR (95% CI)	*P* value
Unadjusted	Average proportion achieving the MHLW reference value (8,000 steps per day) ^c^	0.64 (0.29, 1.40)	0.26
Model 1^a^	Average proportion achieving the MHLW reference value (8,000 steps per day) ^c^	0.32 (0.14, 0.71)	0.01
Model 2^b^	Average proportion achieving the MHLW reference value (8,000 steps per day) ^c^	0.26 (0.11, 0.61)	0.002

**Table 2c:** Hazard ratios by level of daily step count in Japanese office workers (*n* = 730)

Model	Steps	No. of observations	HR (95% CI)	*P* value
Unadjusted	< 5,000	52	ref	
	≥ 5,000 < 8,000	518	0.60 (0.23, 1.56)	0.29
	≥ 8,000 < 10,000	722	0.56 (0.21, 1.47)	0.24
	≥ 10,000 < 12,000	527	0.56 (0.21, 1.46)	0.24
	≥ 12,000	309	0.64 (0.23, 1.76)	0.39
Model 1^a^	< 5,000	52	ref	
	≥ 5,000 < 8,000	518	0.73 (0.27, 1.92)	0.52
	≥ 8,000 < 10,000	722	0.55 (0.21, 1.45)	0.23
	≥ 10,000 < 12,000	527	0.48 (0.18, 1.26)	0.14
	≥ 12,000	309	0.50 (0.18, 1.36)	0.17
Model 2^b^	< 5,000	52	ref	
	≥ 5,000 < 8,000	518	0.48 (0.18, 1.24)	0.13
	≥ 8,000 < 10,000	722	0.39 (0.16, 0.98)	0.05
	≥ 10,000 < 12,000	527	0.30 (0.12, 0.78)	0.01
	≥ 12,000	309	0.34 (0.13, 0.89)	0.03

Hazard ratios (95% confidence intervals) for incidence of metabolic syndrome risk (MetS & pre-MetS). Abbreviations: HR: hazard ratio, CI: confidence interval, MHLW: Ministry of Health, Labour and Welfare, ref: referent

^a^ Model 1 was adjusted for sex and age

^b^ Model 2 was adjusted additionally for average hours of sleep, average frequency of alcohol consumption, smoking status, fast eating, late eating, and willingness to receive health guidance

^c^ Proportion of valid days per year with ≥8,000 steps, averaged per participant across study years

### Sensitivity analysis (alternative MetS classification)

The mean follow-up time was 4.6 (± 1.2) years, with a total of 2,948 person-years. The median (interquartile range) age at AHC 1 was 44 (40–49) years in the < 8,000 steps/day group and 45 (41–51) years in the ≥ 8,000 steps/day group. Table 3a presents the results based on the alternative MetS classification, showing a significant inverse association between daily step count and MetS incidence in all models [Unadjusted: HR = 0.90, 95% CI: 0.83, 0.98; Model 1: HR = 0.84, 95% CI: 0.77, 0.92; Model 2: HR = 0.84, 95% CI: 0.77, 0.92]. Table 3b shows that the current step count reference (8,000 steps/day) is significantly and inversely associated with MetS incidence in both adjusted models, but not in the unadjusted model [Unadjusted: HR = 0.61, 95% CI: 0.27, 1.38; Model 1: HR = 0.32; 95% CI: 0.14, 0.74; Model 2: HR = 0.34; 95% CI: 0.14, 0.83]. Table 3c presents HRs for average daily step count categories compared to the referent < 5,000 steps/day. All step count categories, except for the ≥ 5,000 to 8,000 steps/day group, were significantly and inversely associated with MetS incidence in the fully adjusted model when compared with the referent < 5,000 steps/day [Model 2: ≥5,000 to 8,000: HR = 0.60; 95% CI: 0.21, 1.70; ≥8,000 to 10,000: HR = 0.24; 95% CI: 0.08, 0.68; ≥10,000 to 12,000: HR = 0.21; 95% CI: 0.07, 0.62; ≥12,000: HR = 0.19; 95% CI: 0.06, 0.63, respectively].

**Table 3a:** Hazard ratios for incidence of metabolic syndrome risk in Japanese office workers (*n* = 827)

Model		HR (95% CI)	*P* value
Unadjusted	Average daily Step Count (per 1,000)	0.90 (0.83, 0.98)	0.01
Model 1^a^	Average daily Step Count (per 1,000)	0.84 (0.77, 0.92)	< 0.001
Model 2^b^	Average daily Step Count (per 1,000)	0.84 (0.77, 0.92)	< 0.001

**Table 3b:** Hazard ratios by achievement rate of the MHLW reference value in Japanese office workers (*n* = 827)

Model			HR (95% CI)	*P* value
Unadjusted	Average proportion achieving the MHLW reference value (8,000 steps per day) ^c^		0.61 (0.27, 1.38)	0.24
Model 1^a^	Average proportion achieving the MHLW reference value (8,000 steps per day) ^c^		0.32 (0.14, 0.74)	0.01
Model 2^b^	Average proportion achieving the MHLW reference value (8,000 steps per day) ^c^		0.34 (0.14, 0.83)	0.02

**Table 3c:** Hazard ratios by the category of step count level in Japanese office workers (*n* = 827)

Model	Steps	No. of observations	HR (95% CI)	*P* value
Unadjusted	< 5,000	56	ref	
	≥ 5,000 < 8,000	595	0.83 (0.30, 2.27)	0.71
	≥ 8,000 < 10,000	802	0.42 (0.15, 1.18)	0.10
	≥ 10,000 < 12,000	584	0.43 (0.14, 1.27)	0.13
	≥ 12,000	352	0.40 (0.13, 1.29)	0.13
Model 1^a^	< 5,000	56	ref	
	≥ 5,000 < 8,000	595	0.87 (0.31, 2.41)	0.79
	≥ 8,000 < 10,000	802	0.34 (0.12, 0.96)	0.04
	≥ 10,000 < 12,000	584	0.32 (0.11, 0.94)	0.04
	≥ 12,000	352	0.27 (0.09, 0.87)	0.03
Model 2^b^	< 5,000	56	ref	
	≥ 5,000 < 8,000	595	0.60 (0.21, 1.70)	0.34
	≥ 8,000 < 10,000	802	0.24 (0.08, 0.68)	0.01
	≥ 10,000 < 12,000	584	0.21 (0.07, 0.62)	0.01
	≥ 12,000	352	0.19 (0.06, 0.63)	0.01

Hazard ratios (95% confidence intervals) for incident metabolic syndrome (MetS) based on an alternative classification, among Japanese office workers

Abbreviations: HR: hazard ratio, CI: confidence interval, MHLW: Ministry of Health, Labour and Welfare, ref: referent

^a^ Model 1 was adjusted for sex and age

^b^ Model 2 was adjusted additionally for average hours of sleep, average frequency of alcohol consumption, smoking status, fast eating, late eating, and willingness to receive health guidance

^c^ Proportion of valid days per year with ≥ 8,000 steps, averaged per participant across study years

### Assessment of non-linearity

To evaluate whether the association between step count and MetS incidence was non-linear, a restricted cubic spline model with three knots was compared to the linear model using AIC. The linear model yielded a lower AIC value, indicating a better fit, and was therefore selected for the main analysis.

## Discussion

The results of our analysis indicate that daily step count is significantly and inversely associated with the incidence of MetS in adjusted models. Each additional 1,000 steps per day was associated with a 9% reduction in MetS incidence, while achieving the recommended target of 8,000 steps per day was associated with a 74% reduction in the multivariable-adjusted model. Across step count categories, higher daily step counts were associated with a greater reduction in MetS risk. However, the dose-response effect appeared to plateau beyond 12,000 steps/day for both pre-MetS and MetS. The sensitivity analysis focusing solely on MetS yielded similar results, showing that each additional 1,000 steps per day was associated with a 16% reduction in MetS incidence, and achieving 8,000 steps per day contributed to a 66% reduction in the adjusted model. These findings suggest that daily step count may reduce the risk of MetS in a dose-dependent manner. Although a longer study period would provide a more robust assessment of MetS development, which occurs gradually over time, we employed an interval-censored Cox proportional hazards model to address limitations in the available dataset. These findings may contribute to public health strategies aimed at MetS prevention through habitual daily step accumulation in the general Japanese population.

## Main analysis

Compared with previous studies, and allowing for variability in effect estimates due to differences in study design and populations, our findings are broadly consistent with existing evidence, particularly given that MetS is considered a risk factor and prophase for various chronic diseases. For example, a systematic review by Hall et al. concluded that each 1,000 step/day increment at baseline may reduce the risk of all-cause mortality by 6–36% over 4 to 10 years, and reduce the risk of CVD morbidity and mortality by 5–21% over 2 to 5 years [9]. However, the review did not identify consistent reductions in risk for diabetes or glycemia-related outcomes due to heterogeneity in study populations and analytical approaches. In contrast, Ponsonby et al. conducted a population-based longitudinal study and found that each 1,000-step increment was associated with a 13% reduction in the odds of developing dysglycemia over a 5-year period [28]. Furthermore, a lifestyle intervention study conducted alongside a pharmaceutical clinical trial by Kraus et al. reported that a 2,000-step increment in average daily step reduced the incidence of diabetes by 5.5% among high-risk participants [29].

Regarding the current step count recommendations by the MHLW, our results indicate that achieving 8,000 steps per day was associated with a significant 74% reduction in the risk of MetS in the fully adjusted model (*p* = 0.002). Indeed, when combining the results of analyses using the 8,000 steps/day cutoff with those examining multiple step count categories, a dose-response effect became apparent starting at ≥ 8,000 steps. Moreover, results based on step count categories suggest that exceeding the 8,000 steps/day recommendation may offer additional benefits in reducing MetS risk. Notably, a step count of 10,000 to 12,000 steps/day was associated with a 70% reduction in MetS incidence, while a step count of ≥ 12,000 steps was associated with a 66% reduction. It should be noted that these dose-response findings may lack statistical significance due to overlapping confidence intervals between adjacent step categories. Nonetheless, the results suggest a possible ceiling effect, whereby step counts beyond 12,000 steps/day do not yield additional risk reduction. These trends warrant further investigation to inform step count targets that maximize health benefit. This finding aligns with a population-based study by Cruz et al., which reported that higher daily step counts were associated with reduced all-cause mortality, although the dose-response effect beyond 10,000 steps was less pronounced [12]. The authors speculated that this pattern may be due to the sparsity of data at higher step counts or the increased health consciousness among more active study participants. Similarly, in a longitudinal cohort study of middle-aged Japanese adults, Yamamoto et al. reported a potential non-linear association, with HRs plateauing beyond the 8,000–9,000 steps/day range [7]. In light of these patterns, further investigation of potential non-linear versus linear dose-response associations of step count with MetS risk is warranted in future studies.

Among various recommendations for optimal PA levels in healthy adults [30, 31], the rationale for Japan’s MHLW’s recommendation of 8,000 steps/day, comprising 6,000 steps at ≥ 3 METs and 2,000 habitual steps at < 3 METs, is outlined in the Exercise and Physical Activity Guide [13]. According to Tudor-Locke et al. [30], based on a cadence of 100 steps per minute and sedentary behavior thresholds, a target range of 7,100 − 11,000 steps/day was proposed for promoting health. In light of these step-based threshold, additional studies are warranted to replicate our findings and determine whether the current reference value of 8,000 steps/day may be set too low. Further research should also investigate whether setting a target of ≥ 10,000 step/day would offer greater health benefits while remaining a feasible goal for the general healthy adult population [12, 30]. Using step count as a metric for PA may be preferable for strategy development, although METs, which estimate energy expenditure, may also be used as an alternative [32, 33]. While defining PA indicators in terms of METs is useful for individuals who engage in structured exercise, applying METs to describe common activities such as walking, housecleaning, or gardening can introduce ambiguity. With the advent of technologies enabling objective daily PA measurement, step count offers a clear and unambiguous metric. When harmonized across institutions, it may be especially effective in promoting healthy behaviors and facilitate real-world application [9, 10, 12, 30].

### Comparison of the result with different classification

In the sensitivity analysis, we applied the same analytic models used in the main analysis but adopted an alternative classification of MetS, in which pre-MetS was treated as a healthy status. Although both outcome classifications yielded significant risk reductions, the results differed slightly depending on the definition of MetS used. For example, the association between daily step count and MetS in the main analysis was non-linear, showing a clear ceiling effect, whereas the association in the sensitivity analysis appeared linear. One possible explanation for the discrepancy is that the distribution and frequency of covariates varied slightly depending on the outcome classification. Indeed, Hattori et al. noted that covariates such as lifestyle factors, age cohort, and gender, each of which may influence MetS, can be intricately interrelated [34]. Alternatively, a cross-sectional study by Kim et al., examining the association between PA and MetS (defined as MetS/pre-Mets) in Japanese workers found that the risk of MetS was significantly higher in the physically inactive group (OR = 2.20) compared with the physically active group. However, when stratified by gender, no significant association was observed in women [35]. These findings suggest that heterogeneity arising from classification criteria may have influenced their results. Furthermore, as health organizations employ varying criteria to define MetS [4], studies should clearly specify their operational definitions and target outcomes. In the present study, our primary interest in prevention led us to focus the main analysis on the development of any MetS risk over time in healthy participants, by treating both pre-MetS and MetS as outcome events [7, 35, 36].

### Strengths and limitations

This study has two key strengths. To our knowledge, this is the first study to examine the risk of MetS using a survival model that accounts for its progression from a preventive perspective. It is also the first study to use daily step count data measured by wearable devices under real-world conditions throughout the study period, enabling adjustment for several important covariates. This study also has five limitations: First, some data on important covariates such as alcohol consumption, smoking status, or medication use were missing. Second, the number of participants who developed MetS during the 5-year follow-up period was relatively small, highlighting the need for longer observation periods to assess MetS incidence more robustly. Despite this limitation, several significant associations between daily step count and MetS were observed, suggesting a potentially substantial effect of daily steps on MetS risk. Third, PA intensity could not be used as an exposure, as the dataset lacked information on estimated intensity, activity duration, and PA type (habitual or sports-related activity). Fourth, our analysis of linear associations may have limited power to detect significant differences due to overlapping confidence intervals between adjacent step count categories. Finally, the generalizability of our findings to other ethnic groups may be limited due to the use of Japan-specific MetS diagnostic criteria; however, the results provide important insights for public health policy and practice in Japan.

## Conclusions

Our findings indicate that the risk of developing MetS increases gradually over time and that physical activity, as measured by daily step count, is significantly and inversely associated with MetS incidence in adjusted models. Among generally healthy participants, each additional 1,000 steps per day was associated with a 9% reduction in MetS risk. Participants who averaged 8,000 steps/day, consistent with current Japanese guidelines, were 74% less likely to develop MetS. Moreover, exceeding 8,000 steps/day may provide additional protective benefits. Overall, these results suggest that daily step count is a practical and potentially effective lifestyle factor for MetS prevention. Further research involving more diverse populations, larger sample sizes, and detailed physical activity intensity data is needed to confirm and extend these findings.

## Data Availability

We cannot provide public access to individual data due to participant privacy stipulations in accordance with ethical guidelines. Additionally, the written informed consent we obtained from study participants does not include a provision for the public sharing of data. Qualifying researchers may, upon reasonable request, apply to access an aggregated dataset by contacting the corresponding author.
